# Wide Grain 3, a GRAS Protein, Interacts with DLT to Regulate Grain Size and Brassinosteroid Signaling in Rice

**DOI:** 10.1186/s12284-022-00601-4

**Published:** 2022-11-03

**Authors:** Weilan Chen, Xiaoling Hu, Li Hu, Xinyue Hou, Zhengyan Xu, Fanmin Yang, Min Yuan, Feifan Chen, Yunxiao Wang, Bin Tu, Ting Li, Liangzhu Kang, Shiwen Tang, Bingtian Ma, Yuping Wang, Shigui Li, Peng Qin, Hua Yuan

**Affiliations:** 1 State Key Laboratory of Crop Gene Exploration and Utilization in Southwest China, Rice Research Institute, Sichuan Agricultural University, 611130 Chengdu, Sichuan China; 2 College of Agriculture, Forestry and Health, The Open University of Sichuan, 610073 Chengdu, Sichuan China

**Keywords:** Rice, Grain size, *WG3*, *DLT*, GRAS, Brassinosteroid signaling

## Abstract

**Background:**

Grain size is a direct determinant of grain weight and yield in rice; however, the genetic and molecular mechanisms determining grain size remain largely unknown.

**Findings:**

We identified a mutant, *wide grain 3* (*wg3*), which exhibited significantly increased grain width and 1000-grain weight. Cytological analysis showed that *WG3* regulates grain size by affecting cell proliferation. MutMap-based gene cloning and a transgenic experiment demonstrated that *WG3* encodes a GRAS protein. Moreover, we found that WG3 directly interacts with DWARF AND LOW-TILLERING (DLT), a previously reported GRAS protein, and a genetic experiment demonstrated that WG3 and DLT function in a common pathway to regulate grain size. Additionally, a brassinosteroid (BR) sensitivity test suggested that WG3 has a positive role in BR signaling in rice. Collectively, our results reveal a new genetic and molecular mechanism for the regulation of grain size in rice by the WG3-DLT complex, and highlight the important functions of the GRAS protein complex in plants.

**Conclusion:**

WG3 functions directly in regulating grain size and BR signaling in rice.

**Supplementary Information:**

The online version contains supplementary material available at 10.1186/s12284-022-00601-4.

## Findings

Rice (*Oryza sativa* L.) is a staple food for more than half of the world’s population, and thus increasing rice yield is an important way to ensure food security. Grain size, including grain length, width, and thickness, is a direct determinant of grain weight and yield in rice and an important target trait for breeding selection (Xing et al. 2010; Zuo et al. 2014; Zhao et al. 2022). In recent years, numerous genes related to grain size have been identified. These genes are involved in different regulatory pathways, including G protein signaling pathway, ubiquitin-proteasome degradation pathway, mitogen-activated protein kinase (MAPK) signaling pathway, and as phytohormones, and transcriptional regulators (Zuo et al. 2014; Fan et al. 2019; Li et al. 2019, 2021a). However, the connections between different pathways remains elusive, and more genes need to be identified.

GRAS protein, named from the first three identified members, GIBBERELLIC ACID INSENSITIVE (GAI), REPRESSOR of GAI (RGA), and SCARECROW (SCR) (Pysh et al. 1999), is a large plant-specific gene family. GRAS proteins have a highly conserved C-terminal region, also known as the GRAS domain, and a variable N-terminal region (Pysh et al. 1999), which may confer functional versatility (Sun et al. 2012). GRAS proteins are involved in a variety of biological processes, including gibberellin and BR signal transduction (Ikeda et al. 2001; Tong et al. 2009; Chen et al. 2013), nodulation signaling pathway (Oldroyd et al. 2003), plant growth and development Li et al. 2003, 2021b; Xie et al. 2019; Hughes et al. 2022), and biotic and abiotic stress responses (Ma et al. 2010; Xu et al. 2015; Vleesschauwer et al. 2016; Wang et al. 2021; Lu et al. 2022). A total of 60 GRAS members have been identified in rice (Liu et al. 2014), but only a few have been functionally characterized. For instance, SLENDER RICE 1 (SLR1) and SLR1-like1 (SLRL1) are negative regulators of gibberellin signaling (Ikeda et al. 2001; Itoh et al. 2005); DWARF AND LOW-TILLERING (DLT)/GRAIN SIZE 6 (GS6) positively regulates brassinosteroid (BR) signaling but negatively regulates grain width in rice (Tong et al. 2009; Sun et al. 2013); NODULATION SIGNALING PATHWAY1 (NSP1) and NSP2 regulate strigolactone biosynthesis in rice (Liu et al. 2011); MONOCULM 1 (MOC1) positively controls rice tillering (Li et al. 2003); OsGRAS23 positively modulates rice drought tolerance (Xu et al. 2015); and OsSCL7/OsGRAS19 has positive roles in BR signaling and disease resistance in rice (Chen et al. 2013; Lu et al. 2022). However, the functions of GRAS proteins in regulating plant growth and development, especially grain size in rice, are still largely unknown.

**Fig. 1:**
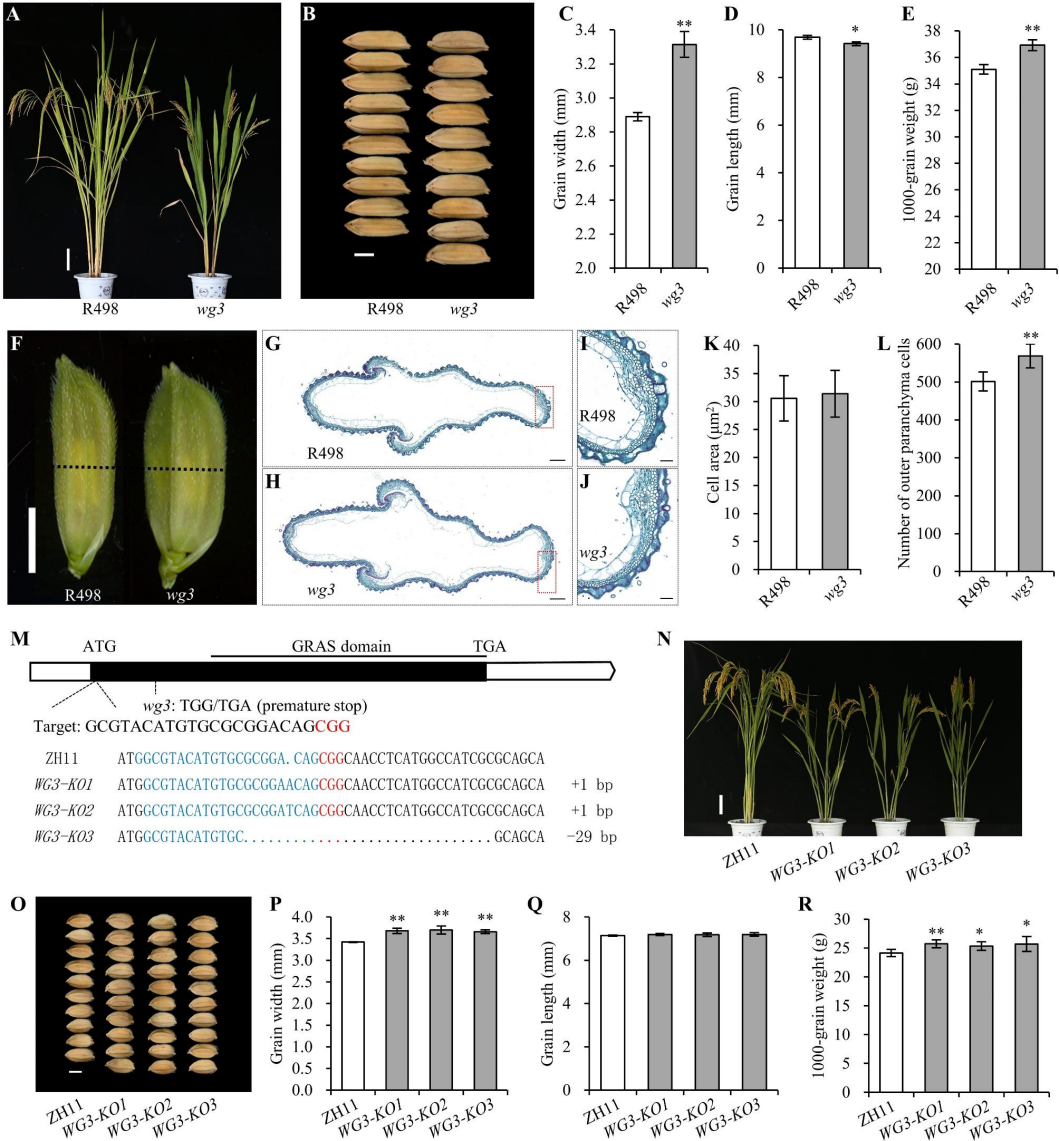
*WG3* regulates grain width by affecting cell proliferation in rice. (A) Plant architecture of R498 and *wg3* at the mature stage. Scale bar, 10 cm. (B) Comparison of grain width between R498 and *wg3*. Scale bar, 3 mm. (C-E) Statistical analysis of grain width (C), grain length (D), and 1000-grain weight (E) between R498 and *wg3*. (F) Spikelet hulls of R498 and *wg3* before flowering. Scale bar, 3 mm. (G, H) Cross-sections of the spikelet hulls of R498 and *wg3*. The position of the cross-section is indicated by a black dotted line in F. Scale bars, 200 μm. (I-J) Magnified views of the cross-section boxes in G and H. Scale bars, 50 μm. (K, L) Comparison of cell area (K) and cell number (L) in the outer parenchyma layer. (M) Schematic diagram of the *WG3* gene, and alignment of *WG3* sequences between ZH11 and *WG3*-*KO* mutants. The GRAS domain, knockout target site, and the mutation site in *wg3* are shown. The PAM sequences are highlighted in red and the target sequences are highlighted in blue. (N) Plant architecture of ZH11 and *WG3*-*KO* mutants at the mature stage. Scale bar, 10 cm. (O) Comparison of grain width between ZH11 and *WG3*-*KO* mutants. Scale bar, 3 mm. (P-R) Statistical analysis of grain width (P), grain length (Q), and 1000-grain weight (R) between ZH11 and *WG3*-*KO* mutants. Data are given as means ± SD [*n* = 3 replicates, and each replicate contained five plants in (C-E); *n* = 20 in (K, L); *n* = 6 plants in (P-R)]. Significant differences between wild type and corresponding mutants (*P < 0.05 and **P < 0.01) were determined using a Student’s *t*-test

In this study, we identified a *wide grain 3* (*wg3*) mutant from the ethyl methanesulfonate mutant library of the *indica* restorer line Shuhui 498 (R498) (Fig. 1a). Compared with R498, the grain width of *wg3* significantly increased by 14.65%, and although the grain length was slightly but significantly decreased, the final 1000-grain weight was increased (Fig. 1b-e). In general, grain size is coordinately controlled by cell proliferation and cell expansion in the spikelet hull (Li et al. 2018). We investigated the cell size and number in spikelet hulls by paraffin section, and found that the cell size in *wg3* was not significantly different compared with R498, but the cell number in the transverse direction was significantly increased in *wg3* (Fig. 1f-l). Additionally, we compared the expression levels of 26 cell-cycle related genes between R498 and *wg3*, including 18 putatively involved in the G1/S phase and eight in the G2/M phase, and found that most of the genes involved in the G1/S phase were significantly up-regulated in the *wg3* mutant (Additional file 1: Figure S1). These results indicated that *WG3* regulates grain size by affecting cell proliferation.

To identify the causal gene responsible for wide grains of *wg3* mutants, we generated an F_2_ population by crossing *wg3* with its wild-type plant R498. There were 86 plants with wide grains, and 325 plants with normal grains, conforming to a 1:3 segregation ratio (χ^2^_c_ = 3.43 < χ^2^_0.05(1)_ = 3.84) (Additional file 1: Figure S2a), indicating that one recessive locus was responsible for the wide grains of *wg3*. We then identified the *wg3* mutation using the MutMap strategy (Abe et al. 2012). Two mutation sites with Euclidean distance (ED^4^) of 4 were identified by alignment with the reference genome *Nipponbare*; however, mutation 2 (M2) was not different between the normal grain and wide grain bulks, and only M1 located in the coding region of *LOC_Os03g51330* were co-segregated with the wide grain phenotype (Additional file 1: Figure S2b-d); M1 leads to a stop codon (Fig. 1 m; Additional file 1: Figure S3). *LOC_Os03g51330*, encoding a GRAS protein, has a relatively high expression level in developing young panicles (Fig. 1 m; Additional file 1: Figure S4). Therefore, mutation in *LOC_Os03g51330* was likely responsible for the wide grain of *wg3*, hereafter, *LOC_Os03g51330* was named *WG3*.

To further confirm the function of *WG3*, we generated knockout (KO) mutants of *WG3* in the *japonica* cultivar Zhonghua 11 (ZH11) background using the CRISPR/Cas9 genome editing system (Ma et al. 2015). Three independent homozygous *WG3-KO* lines were obtained (Fig. 1 m, n), and all the *KO* lines harbored a truncated protein of WG3 without the conserved GRAS domain (Additional file 1: Figure S3). As expected, *WG3-KO* lines showed significantly increased grain width and 1000-grain weight, though the grain length showed no significant difference (Fig. 1o-r). Taken together, these results demonstrated that *WG3* is essential in regulating grain size and grain weight in rice.

**Fig. 2:**
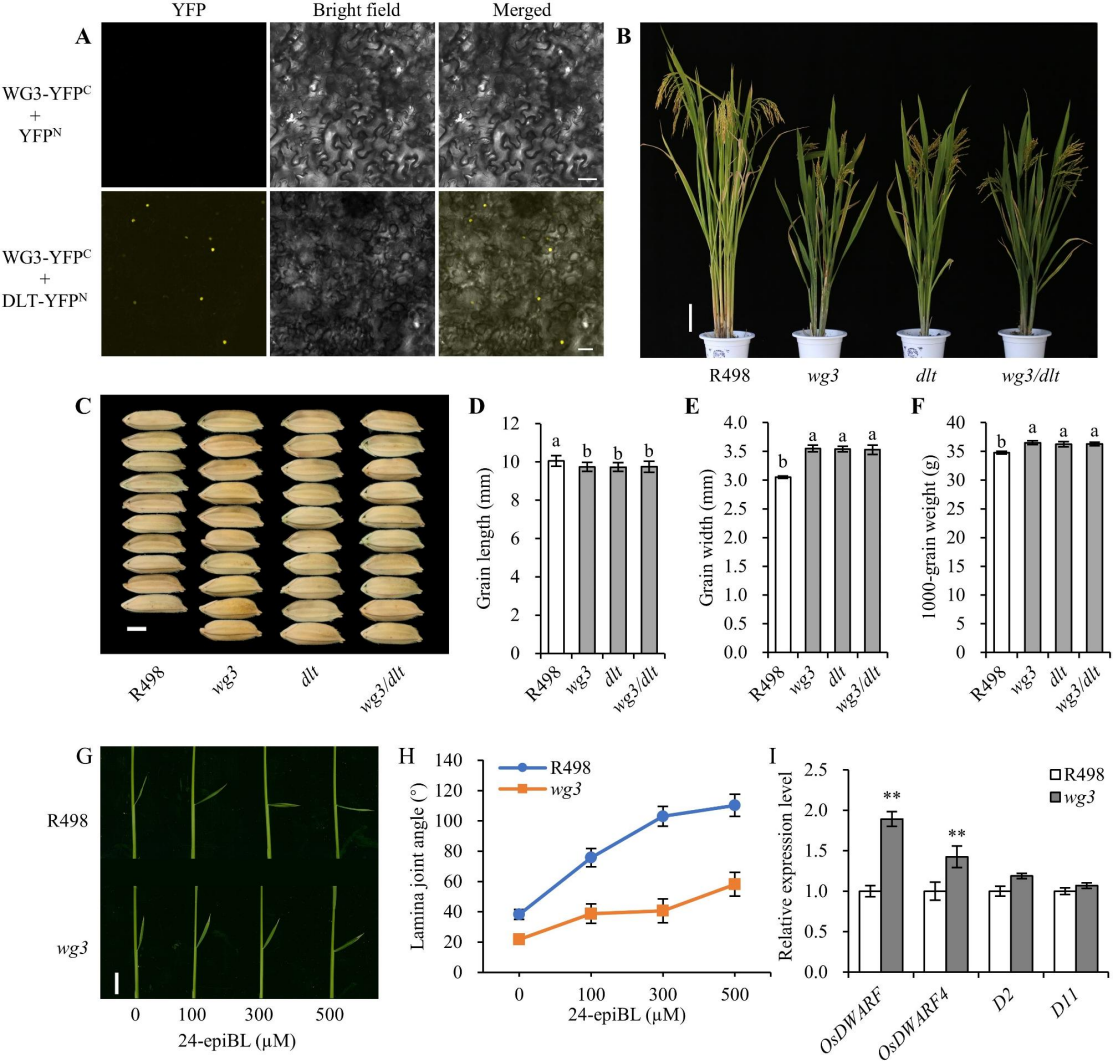
WG3 interacts with DLT to regulate grain size and BR signaling in rice. (A) BiFC analysis of WG3 and DLT interaction in the epidermal cells of *Nicotiana benthamiana* leaves. Scale bar, 50 μm. (B) Plant architecture of R498, *wg3*, *dlt*, and *wg3*/*dlt* at the mature stage. Scale bar, 10 cm. (C) Comparison of grain width between R498, *wg3*, *dlt*, and *wg3*/*dlt*. Scale bar, 3 mm. (D-F) Statistical analysis of grain length (D), grain width (E), and 1000-grain weight (F) between R498, *wg3*, *dlt*, and *wg3*/*dlt*. Data are means ± SD (*n* = 3 replicates). Significant differences between R498, *wg3*, *dlt*, and *wg3*/*dlt* were determined using Duncan’s multiple comparisons. (G) Lamina inclination of R498 and *wg3* plants treated with different concentrations of 24-epiBL. Scale bar, 1 cm. (H) Statistical analysis of lamina joint angle in G. Data are means ± SD (*n* = 20 plants). (I) Comparison of expression levels of four BR biosynthesis genes (*OsDWARF*, *OsDWARF4*, *D2*, and D11) in the young panicles of R498 and *wg3*. *UBQ5* and *FhaB* were used as internal controls, and the values of R498 were set to 1. Data are means ± SD (*n* = 3 replicates). Significant differences between R498 and *wg3* (**P < 0.01) were determined using Student’s *t*-test

In addition to grain size, we noted that both *wg3* mutant and *WG3-KO* lines showed other phenotypic differences, including decreased plant height, tiller number, panicle length, and seed setting rate (Additional file 1: Table S1), which are reminiscent of the phenotypes in the *dlt* mutant (Tong et al. 2009). Considering that GRAS proteins can function as heterodimers (Cui et al. 2007; Hirsch et al. 2009), we wondered whether these two GRAS proteins, WG3 and DLT, could also form a complex. To test this hypothesis, we performed a bimolecular fluorescence complementation (BiFC) assay, and observed unambiguous nuclear localization of YFP fluorescence signals when WG3-YFP^C^ was co-expressed with DLT-YFP^N^ in leaf epidermal cells of *Nicotiana benthamiana* (Fig. 2a), indicating that WG3 can interact with DLT in plant cells.

Coincidentally, we identified another mutant with phenotypes similar to those of *wg3* in the same R498 mutant library, including decreased plant height and tiller number, and increased grain width and 1000-grain weight (Additional file 1: Figure S5a-e), but which was not allelic to *wg3*. MutMap and co-separation analysis demonstrated that a 50-bp deletion located in the exon of *DLT* (*LOC_Os06g03710*), which led to a premature stop code and truncated GRAS domain (Additional file 1: Figure S5f-i), was responsible for these phenotypes. Thereafter, we named this mutant *dlt*. To further analyze the genetic relationship between WG3 and DLT, we generated a double mutant, *wg3/dlt*, by crossing *wg3* with *dlt* single mutant (Fig. 2b). The phenotypes of the double mutant, including plant architecture and grain size, were indistinguishable from those in *wg3* and *dlt* single mutants (Fig. 2b-f; Additional file 1: Figure S6). These results suggested that WG3 and DLT function in a common genetic pathway to regulate plant architecture and grain size in rice.

Considering that DLT is a positive regulator of BR signaling (Tong et al. 2009), and WG3 functions in a common pathway with DLT (Fig. 2b-f), we performed a BR-induced lamina inclination assay to investigate whether WG3 is involved in BR signaling in rice. As expected, the lamina joint angle of wild-type R498 was significantly increased with 24-epibrassinolide (24-epiBL) treatment in a dose-dependent manner, however, the *wg3* mutant showed greatly reduced sensitivity to 24-epiBL treatment (Fig. 2g-h). It is well known that BR-signaling related mutants usually initiate feedback regulation of BR biosynthetic genes (Tong et al. 2009; Yuan et al. 2017, 2022). Because of this, we investigated the expression levels of four BR biosynthetic genes in the young panicles of R498 and *wg3*, and found that two of them (*OsDWARF* and *OsDWARF4*) were significantly up-regulated in *wg3* (Fig. 2i). This indicated that the feedback regulation of BR biosynthesis genes was activated in *wg3* plants. These results suggested that WG3 is positively involved in BR signaling in rice.

Taken together, we identified a new grain size related gene, *WG3*, which encodes a GRAS protein. *WG3* regulates grain size by affecting cell proliferation. Importantly, WG3 directly interacts with DLT, and they regulate grain size in a common genetic pathway. Additionally, *WG3* positively participates in BR signaling in rice. Thus, our results highlight the important roles of the GRAS protein complex in regulation of grain size and BR signaling in rice.

## Electronic Supplementary Material

Below is the link to the electronic supplementary material.

**Additional file 1:Figure S1.** Comparison of the expression levels of cell-cycle related genes in young panicles of R498 and *wg3* by qRT–PCR. **Figure S2.** Identification of the causal gene responsible for wide grains of the *wg3* mutant using the MutMap strategy. **Figure S3.** Alignment of the amino acid sequences of WG3 from WT and mutants. **Figure S4.** Expression pattern analysis of *WG3*. **Figure S5.** A *dlt* mutant was identified from the R498 mutant library using the MutMap strategy. **Figure S6.** Plant architecture related traits of R498, *wg3*, *dlt*, and *wg3*/*dlt* plants. **Table S1.** Agronomic traits of R498, *wg3*, ZH11, and *WG3-KO* mutants. **Table S2.** Primers used for qRT–PCR analysis in this study. **Table S3.** Primers used for PCR amplification and plasmid construction.

**Additional file 2:** Materials and Methods.

## Data Availability

All data generated or analyzed during this study are included in this published article and its supplementary information files.

## Abbreviations

WG3: Wide Grain 3
DLT: Dwarf and low-tillering
R498: Shuhui 498
ZH11: Zhonghua 11
MAPK: Mitogen-activated protein kinase
BR: Brassinosteroid
CRISPR: Clustered regularly interspaced short palindromic repeats
KO: Knockout
BiFC: Bimolecular fluorescence complementation
YFP: Yellow fluorescent protein
24-epiBL: 24-epibrassinolide
