# Supplementary material for: Wide Grain 3, a GRAS Protein, Interacts with DLT to Regulate Grain Size and Brassinosteroid Signaling in Rice
Source: Rice (N Y). 2022 Nov 3;15:55. doi: 10.1186/s12284-022-00601-4 (PMC9633911; doi:10.1186/s12284-022-00601-4)
Supplement: Supplementary file 1 — Additional file 1:Figure S1. Comparison of the expression levels of cell-cycle related genes in young panicles of R498 and wg3 by qRT–PCR. Figure S2. Identification of the causal gene responsible for wide grains of the wg3 mutant using the MutMap strategy. Figure S3. Alignment of the amino acid sequences of WG3 from WT and mutants. Figure S4. Expression pattern analysis of WG3. Figure S5. A dlt mutant was identified from the R498 mutant library using the MutMap strategy. Figure S6. Plant architecture related traits of R498, wg3, dlt, and wg3/dlt plants. Table S1. Agronomic traits of R498, wg3, ZH11, and WG3-KO mutants. Table S2. Primers used for qRT–PCR analysis in this study. Table S3. Primers used for PCR amplification and plasmid construction. [file 12284_2022_601_MOESM1_ESM.pdf]

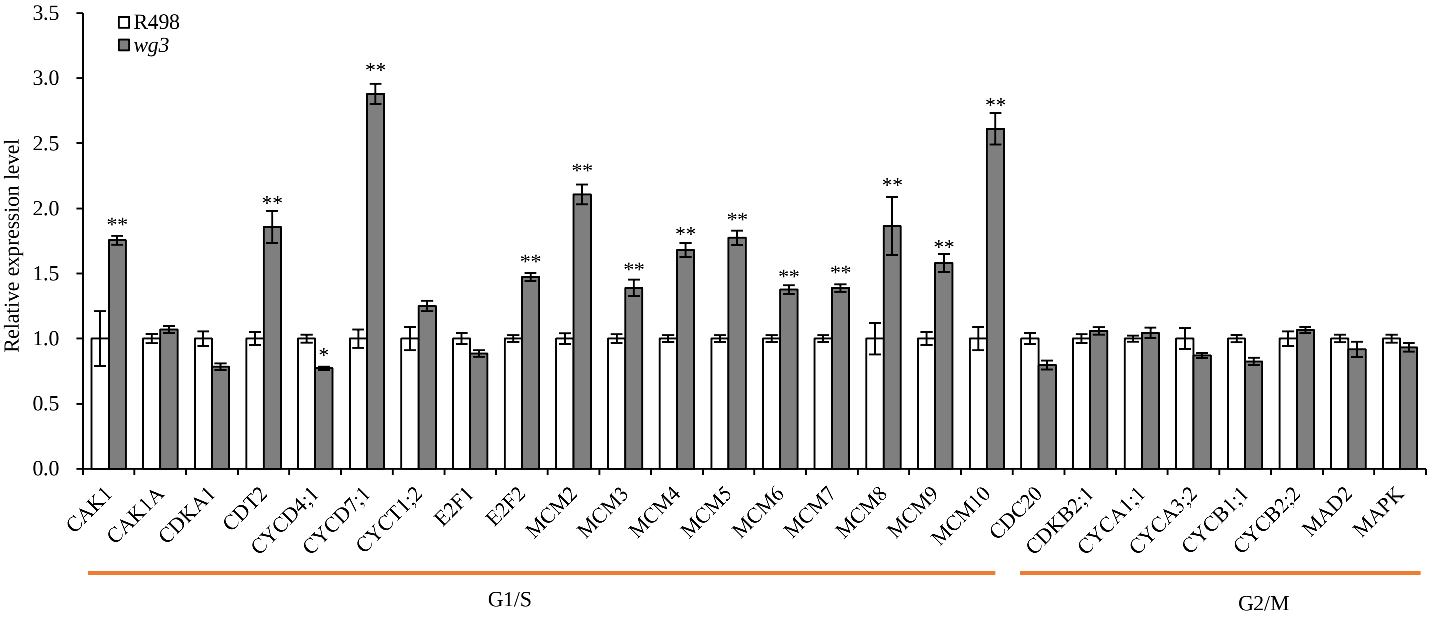

Figure S1. Comparison of the expression levels of cell-cycle related genes in young panicles of R498 and *wg3* by qRT–PCR. *UBQ5* and *FhaB* were used as internal controls, and the values of R498 were set to 1. Data are given as means ±SD (*n*=3 replicates). Significant differences between R498 and *wg3* (*P<0.05 and **P<0.01) were determined using a Student's *t*-test.

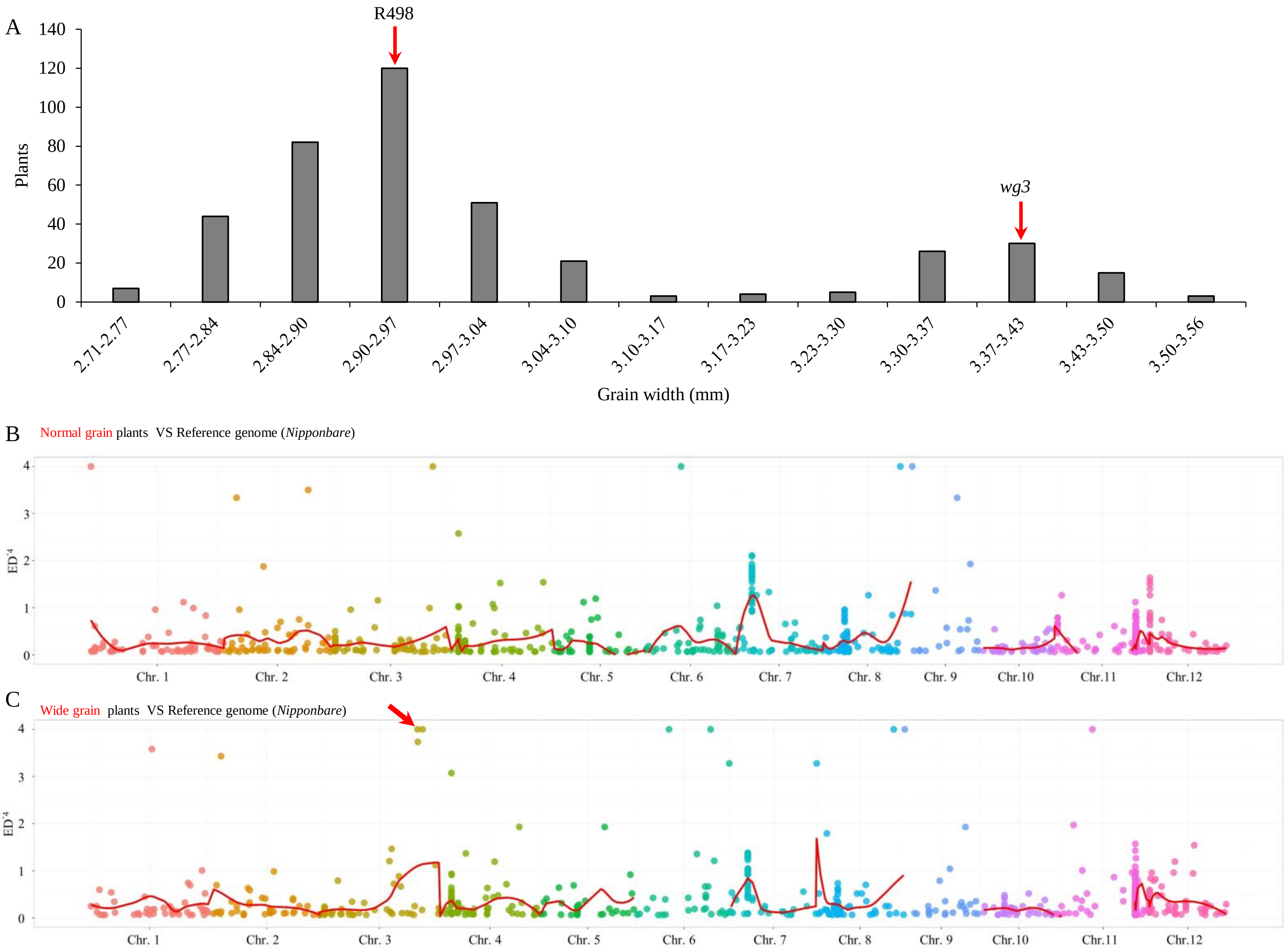

D

No.	Chr.	Position	Reference (*Nipponbare*)	Normal grain	Wide grain	ED^4	Gene ID	Annotation
M1	3	32373168	G	G/A; 24,11	A/A; 0,31	4	LOC_Os03g51330	Stop gained
M2	3	34063843	A	AATCTATAGATAGAAATAAGGCAACTAT/ AATCTATAGATAGAAATAAGGCAACTAT; 0,13	AATCTATAGATAGAAATAAGGCAACTAT/ AATCTATAGATAGAAATAAGGCAACTAT; 0,18	4	LOC_Os03g53990	Downstream

Figure S2. Identification of the causal gene responsible for wide grains of the *wg3* mutant using the MutMap strategy.

(A) Distribution of grain width in the F_2 population (n=411) derived from a cross between R498 and *wg3*. Arrows indicate the mean grain widths of R498 and *wg3* plants. (B-C) Euclidean distance (ED) scores across the genome. Arrow indicates the linkage mutations on chromosome 3. (D) Two mutations with ED^4 of 4 were identified on chromosome 3.

```
R498        MAYMCADSGNLMAIAQQVIQQQQQQQQQQQRHHHHHHLPPPPPPQSMAPHHHQQKHHHHHQQMPAMPQAPHSSHGQIPGQLAYGGGAAWPAGEHFFADAF   100
wg3         MAYMCADSGNLMAIAQQVIQQQQQQQQQQQRHHHHHHLPPPPPPQSMAPHHHQQKHHHHHQQMPAMPQAPHSSHGQIPGQLAYGGGAA*...........    88
ZH11        MAYMCADSGNLMAIAQQVIQQQQQQQQQQQRHHHHHHLPPPPPPQSMAPHHHQQKHHHHHQQMPAMPQAPPSSHGQIPGQLAYGGGAAWPAGEHFFADAF   100
WG3-KO1     MAYMCAeqrqphghraagdpaaaaaataaaeapppppsaaaaaaavdgaapppaeappppadagdaagaalvarpdPGparvrwrrgvarrgallrgrv   100
WG3-KO2     MAYMCADqrqphghraagdpaaaaaataaaeapppppsaaaaaaavdgaapppaeappppadagdaagaalvarpdPGparvrwrrgvarrgallrgrv   100
WG3-KO3     MAYMCAagdpaaAaAataaaeapppppsaaaaaaavdgaaPPPaeappPppadagdaagaalvarpdpgParvrwrrgvarrgallrgrvrGvrrrrgvl   100

R498        GASAGDAVFSDLAAAADFDSDGWMESLIGDAPFQDSDLERLIFTTPPPPVPSPPPTHAAATATATAATAAPRPEAAPALLPQPAAATPVACSSPSPSSAD   200
wg3         ....................................................................................................    88
ZH11        GASAGDAVFSDLAAAADFDSDGWMESLIGDAPFQDSDLERLIFTTPPPPVPSPPPTHAAATATATAATAAPRPEAAPALLPQPAAATPVACSSPSPSSAD   200
WG3-KO1     rgvrrrrgvlrprggrglrl*...............................................................................   120
WG3-KO2     rgvrrrrgvlrprggrglrl*...............................................................................   120
WG3-KO3     rprgGrglrl*.........................................................................................   110

R498        ASCSAPILQSLLSCSRAAATDPGLAAAELASVRAAATDAGDPSERLAFYFADALSRRLACGTGAPPSAEPDARFASDELTLCYKTLNDACPYSKFAHLTA   300
wg3         ....................................................................................................    88
ZH11        ASCSAPILQSLLSCSRAAATDPGLAAAELASVRAAATDAGDPSERLAFYFADALSRRLACGTGAPPSAEPDARFASDELTLCYKTLNDACPYSKFAHLTA   300
WG3-KO1     ....................................................................................................   120
WG3-KO2     ....................................................................................................   120
WG3-KO3     ....................................................................................................   110

R498        NQAILEATGAATKIHIVDFGIVQGIQWAALLQALATRPEGKPTRIRITGVPSPLLGPQPAASLAATNTRLRDFAKLLGVDFEFVPLLRPVHELNKSDFLV   400
wg3         ....................................................................................................    88
ZH11        NQAILEATGAATKIHIVDFGIVQGIQWAALLQALATRPEGKPTRIRITGVPSPLLGPQPAASLAATNTRLRDFAKLLGVDFEFVPLLRPVHELNKSDFLV   400
WG3-KO1     ....................................................................................................   120
WG3-KO2     ....................................................................................................   120
WG3-KO3     ....................................................................................................   110

R498        EPDEAVAVNFMLQLYHLLGDSDELVRRVLRLAKSLSPAVVTLGEYEVSLNRAGFVDRFANALSYYRSLFESLDVAMTRDSPERVRVERWMFGERIQRAVG   500
wg3         ....................................................................................................    88
ZH11        EPDEAVAVNFMLQLYHLLGDSDELVRRVLRLAKSLSPAVVTLGEYEVSLNRAGFVDRFANALSYYRSLFESLDVAMTRDSPERVRVERWMFGERIQRAVG   500
WG3-KO1     ....................................................................................................   120
WG3-KO2     ....................................................................................................   120
WG3-KO3     ....................................................................................................   110

R498        PEEGADRTERMAGSSEWQTLMEWCGFEPVPLSNYARSQADLLLWNYDSKYKYSLVELPPAFLSLAWEKRPLLTVSAWR*                        578
wg3         ..............................................................................                          88
ZH11        PEEGADRTERMAGSSEWQTLMEWCGFEPVPLSNYARSQADLLLWNYDSKYKYSLVELPPAFLSLAWEKRPLLTVSAWR*                        578
WG3-KO1     ..............................................................................                         120
WG3-KO2     ..............................................................................                         120
WG3-KO3     ..............................................................................                         110
```

Figure S3. Alignment of the amino acid sequences of WG3 from WT and mutants. The putative GRAS domain, which was identified using SMART (http://smart.embl-heidelberg.de/), is highlighted in green. The sequences of the *KO* mutant proteins that differ from the WT proteins are marked in red.

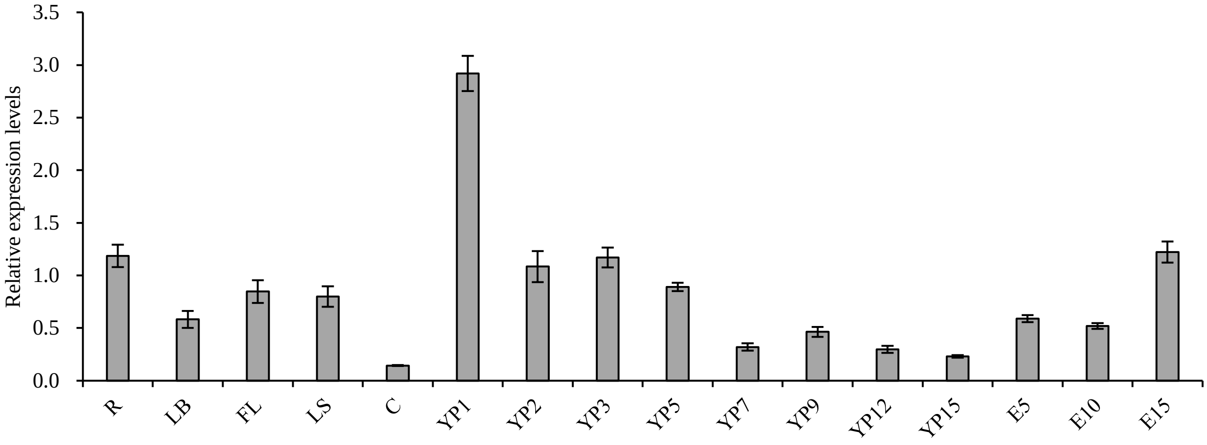

Figure S4. Expression pattern analysis of *WG3*.

R and LB indicate root and leaf blade, respectively, at the seedling stage; FL, LS, and C indicate flag leaf, leaf sheath, and culm, respectively, at the booting stage; YP1-YP15 indicate young panicles with different lengths (cm); E5, E10, and E15 indicate endosperm 5, 10 and 15 d after fertilization, respectively. Data are given as means ±SD (*n*=3 replicates).

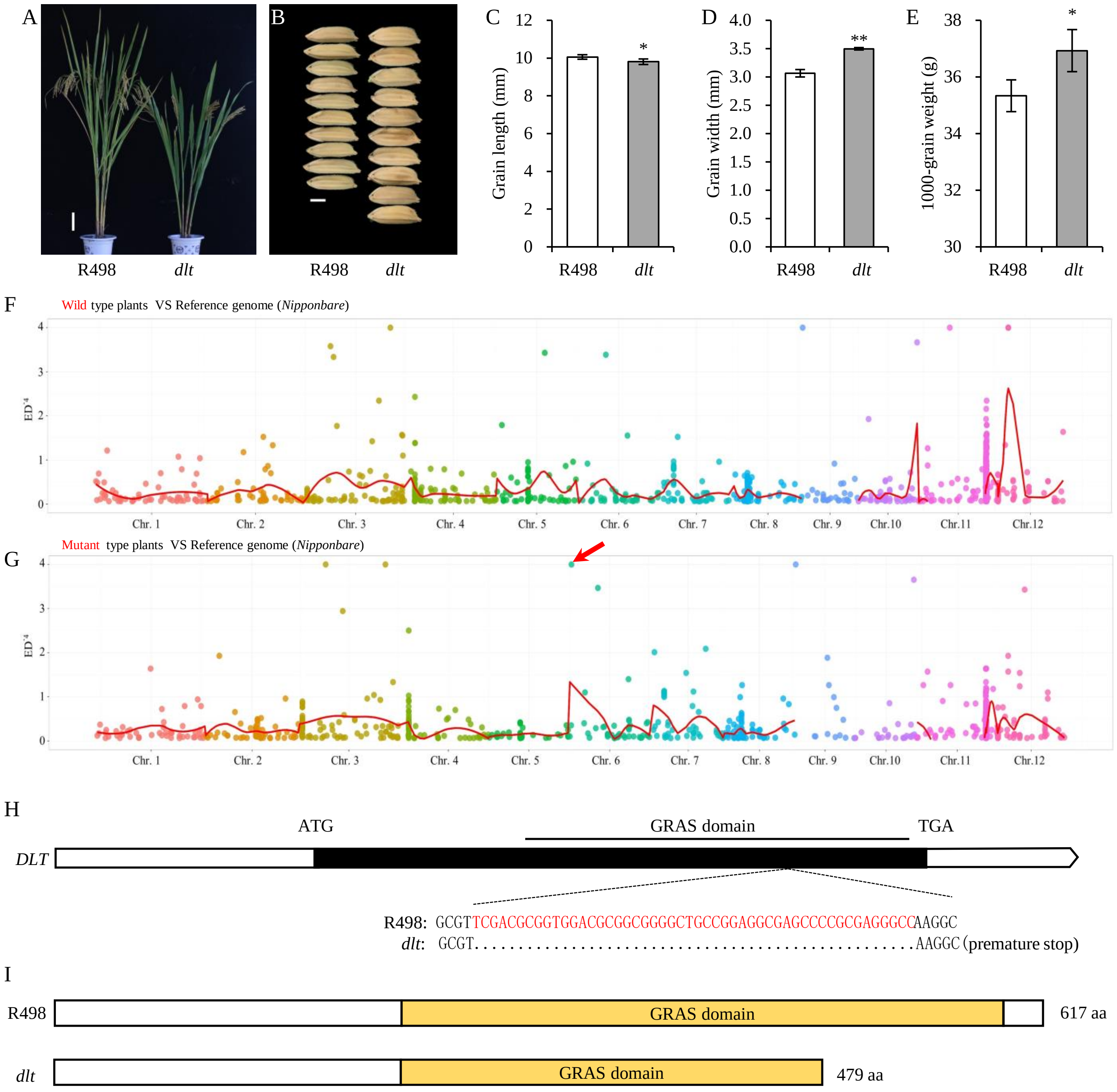

Figure S5. A *dlt* mutant was identified from the R498 mutant library using the MutMap strategy.

(A) Plant architecture of R498 and *dlt* at the mature stage. Scale bar, 10 cm. (B) Comparison of grain widths between R498 and *dlt*. Scale bar, 3 mm. (C-E) Statistical analysis of grain length (C), grain width (D), and 1000-grain weight (E) between R498 and *dlt*. Data are given as means ±SD (*n*=3 replicates). Significant differences between R498 and *dlt* (**P*<0.05 and ***P*<0.01) were determined using a Student's *t*-test. (F-G) ED scores across the genome. Arrow indicates the linkage mutation on chromosome 6. (H) Schematic diagram of the *DLT* gene. The GRAS domain and the mutation site in *dlt* are shown. (I) Mutation in *dlt* leads to a truncated GRAS domain of DLT.

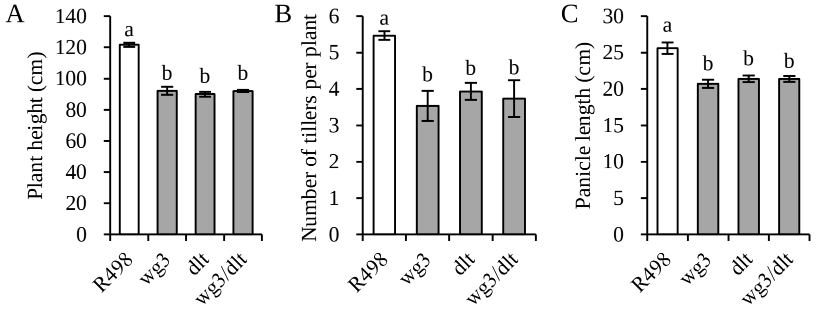

Figure S6. Plant architecture related traits of R498, *wg3*, *dlt*, and *wg3*/*dlt* plants.

(A-C) Statistical analysis of plant height (A), tiller number per plant (B), and panicle length (C) of R498, *wg3*, *dlt*, and *wg3*/*dlt*. Data are means ±SD (*n*=3 replicates). Significant differences between R498, *wg3*, *dlt*, and *wg3*/*dlt* were determined by Duncan's multiple comparisons.

Table S1. Agronomic traits of R498, *wg3*, ZH11 and *WG3-KO* mutants

Trait	R498	*wg3*	ZH11	*WG3-KO1*	*WG3-KO2*	*WG3-KO3*
Grain width (mm)	2.89±0.03	3.31±0.09**	3.42±0.01	3.68±0.06**	3.70±0.09**	3.66±0.04**
Grain length (mm)	9.69±0.10	9.42±0.10*	7.14±0.03	7.19±0.05	7.18±0.08	7.19±0.08
1000-grain weight (g)	35.09±0.45	36.92±0.51**	24.13±0.60	25.75±0.70**	25.34±0.74*	25.70±1.30*
Plant height (cm)	118.92±0.95	90.13±1.85**	117.42±3.64	78.08±0.66**	77.17±1.03**	78.33±1.86**
Number of tillers per plant	6.07±0.42	4.00±0.20**	7.83±0.98	4.67±0.52**	4.17±0.98**	4.50±1.22**
Panicle length (cm)	25.79±0.20	19.97±0.70**	22.63±0.15	18.03±0.63**	18.40±0.49**	18.53±0.12**
Number of grains per panicle	196.32±10.10	171.52±13.42	184.10±18.22	166.81±10.42	165.99±20.03	172.82±4.61
Seed setting rate (%)	88.08±1.31	75.51±3.66**	85.21±1.69	76.25±2.43**	79.57±3.95**	78.73±2.87**
Grain yield per plant (g)	36.81±3.11	19.06±0.32**	29.77±3.75	14.18±2.99**	14.91±3.82**	15.80±3.18**

Significant differences were determined using a Student's *t*-test between the mutants and the corresponding wild type plants. * and ** indicate $P<0.05$ and $P<0.01$, respectivly.

Table S2. Primers used for qRT–PCR analysis in this study.

Gene	Forward primer(5'-3')	Reverse primer(5'-3')
UBQ5	ACCTTCATGGCCAACCACTT	CTAAGCCTGCTGGTTGTAGA
FhaB	GAGAAGCAGAAGCAGAAGGA	CTTGAATAGGACCATCAGGC
WG3	AACAAGTCGGATTTCTTGGTTG	GACGAAGCACTCTTCTCACTAG
CAK1	GACGGTCAGATTAGACGCAAGA	TCCAAAGGATGTCCACA
CAK1A	GACCGACAAGGGTTTCAGCAT	CCAGCATGTTCAGGAAGATACAAT
CDKA1	GGTTTGGACCTTCTCTCTAAAATGC	AGAGCCTGTCTAGCTGTGATCCTT
CDT2	AACCGCACCAAACACTGGAA	GCAATTCACCATCTGCACTGG
CYCD4;1	GCCATGGAGTTGATACATCCAA	CCAGTAGGGCTCCGTGGAAT
CYCD7;1	CCTTCCACACTGACGGTACAGTT	TGCCGCTGCCAAATAGACA
CYCT1;2	GCATTTGTTGCAGCTCAAG	TCACCACTTCGCTGACTTATTG
E2F1	GGTTGGGCAATGTAGCAACT	AATGCTGACATCCCCTTCTG
E2F2	TGTTGGTGGCTGCCGATAT	CGCCAGGTGCACCCTTT
MCM10	GTTCCACCAATCCACAGA	CAGCGTCTTAGCACTATACT
MCM2	AAGTTGGCAAAAGATCCACGG	CCCCCAAACATAGCTAGTGCAA
MCM3	TTCATGCGTCACTAAATGCGAG	TGAATCTGGAAGCCCAATGTTC
MCM4	CCCGAATGCGATTCTCTGAA	ACCAGTGGCATGATCAGTTGC
MCM5	AAGGAGAACTGCCTGTCCATGA	AGTGGCCTTAGCTTTCACCCTC
MCM6	ATGCTGCTCCTCAACAAG	TGCTGCCGTAGTCTCATA
MCM7	GTTCTCTGACTGTTCATCTTAG	GGTGATAGACATTGATTCCAAG
MCM8	GACGGAATCCTTATATGACAAG	GACGATACCTGATACATTGATG
MCM9	CCAATGAACTGAAGTCTGATCT	CCAATTAAGGTAAGAGCAACTG
CDC20	TCGAATCACCTGTTTGTTGGC	TGGAGACAATCCAACGCAAAG
CDKB2;1	AAGTTTGGCCAGGAGTGAGCA	TCAAGAGCATCAGCGTCGAGA
CYCA1;1	GTTTCGGTTGACGAGACGATGT	CGCTGCAAGGAACCTAGAACTG
CYCA3;2	AGGTTGTCAAGATGGAGAGCGA	CGCTTTTTGTCTTCCTGGCA
CYCB1;1	GGATTTACCGAGTCACAGCT	CAAGCGTGTCAAGATTCCAA
CYCB2;2	CTCAAGGCTGCACAATCTGACA	GCATTGACGGCTGGAATTTG
MAD2	GAGCCATGCATATTCGACGTG	GGTGTCGAAGGAATGCAGCTT
MAPK	ACAGAGCAGCCGAATTTTGAGA	TTCAGCGAAGCTCACACTTGG
OsDWARF	TCAACCTTCCTGGAACCAAC	TCTGTGAGCTTCTCCCTGGT
OsDWARF4	GAGATGGTTTTCACGCAATGTG	ACCCTTGTAGTGCACGTCCTTG
D2	TCGGTGGTGTTCGAAATCCT	ATGAGGCCGACAATAAATTCCT
D11	TGGCGATGACATTCCGATG	GCAACTGCAAACCTGTCAGGA

Table S3. Primers used for PCR amplification and plasmid constructions.

Primer name	Primer sequence (5'-3')	Purpose	Plasmid
Y2274	CCATCACGCCCATCCCGAGGTT	Identification the dlt mutant	/
Y2275	TGCTGTTGTGGACGCCGACACG		
Y2276	ACCACCGTTCGACGAGTTTCTC	Identification the wg3 mutant	/
Y2277	ATGGCCTGGTTGGCGGTGAGGTGCG		
Y2268	CCCCACGCCCTACTGTTTTA	Identification the knockout mutants of *WG3*	/
Y2269	GTCCGCGAAGAAGTGCTCCC		
Y3328-F	gagaacacgggggactctagaATGGCGTACATGTGCGCG	Bimolecular fluorescence complementation assays for detecting protein interactions	35S::WG3-YFP^{N}
Y3329-R	atcgtatgggtacatcccgggCCTCCAAGCAGACACAGTAAGCA		
Y3330-F	gagaacacgggggactctagaATGTTGGCGGGTTGCTCG		35S::DLT-YFP^{C}
Y3331-R	caacttttgctccatcccgggGCTTTGCTGAGAATGTGATGCTG		
Y2112	gccgCGTACATGTGCGCGGACAG	Gene editing	CRISPR/Cas9 plasmid construct for *WG3*
Y2113	aaacCTGTCCGCGCACATGTACG		
