# Supplementary material for: Wide Grain 3, a GRAS Protein, Interacts with DLT to Regulate Grain Size and Brassinosteroid Signaling in Rice
Source: Rice (N Y). 2022 Nov 3;15:55. doi: 10.1186/s12284-022-00601-4 (PMC9633911; doi:10.1186/s12284-022-00601-4)
Supplement: Supplementary file 2 — Additional file 2: Materials and Methods. [file 12284_2022_601_MOESM2_ESM.docx]

**Materials and Methods**

**Plant materials and growth conditions**

The *wg3* and *dlt* mutants were obtained from an ethyl methanesulfonate mutant library of the *Oryza sativa* L. *indica* cultivar Shuhui498 (R498), and a double mutant, *wg3/dlt*, was generated by crossing *wg3* with *dlt*. *Japonica* cultivar Zhonghua 11 (ZH11) was used for transformation. All plants for agronomic trait investigations were grown in the experimental field of Sichuan Agricultural University (Wenjiang, Sichuan, China) under natural conditions. *Nicotiana benthamiana* used for the bimolecular fluorescence complementation (BiFC) assay and rice seedlings used for the lamina inclination assay were grown under the same conditions as previously described (Yuan et al. 2022b).

**Measurement of agronomic traits and statistical analysis**

To investigate the phenotypes of *wg3*, a series of agronomic traits were measured according to our previous descriptions (Yuan et al. 2022a). The grain size and 1000-grain weight were measured with a Mini 1600 automatic analysis system (Jie Lai Mei Technology Co. Ltd., Chengdu, China). The mean value, standard deviation (SD), and Student’s *t*-test were determined using Microsoft Excel 2019 (Microsoft, Redmond, USA). Duncan’s multiple comparisons were conducted using Data Processing System (Tang et al. 2013).

**Paraffin section**

Spikelet hulls from R498 and *wg3* were collected before anthesis and fixed in FAA solution (50% ethanol, 5% glacial acetic acid, and 3.7% formaldehyde) for more than 24 h. The fixed samples were dehydrated in a graded ethanol series (from 30**–**100%), and cleared in a xylene series (50%, 75%, 100%, and 100%). Then, the samples were embedded in Paraplast Plus (Sigma, USA) and cut into sections using a rotary microtome (Leica RM2016, Germany), followed by toluidine blue staining for light microscopy (NIKON ECLIPSE CI, Japan). Cell area was measured using Image-Pro Plus 6.0 (Media Cybernetics, USA).

**MutMap analysis**

For gene mapping, we generated an F_2_ population from a cross between the *wg3* mutant and the wild-type R498. Twenty-five plants with extremely wide grains (WG) as *wg3* and with normal grains (NG) as R498 were respectively selected from the F_2_ population, and genomic DNA of each selected plant was extracted and bulked in equal proportions to generate WG and NG bulks. Each DNA bulk was then subjected to whole-genome resequencing with an MGISEQ-2000 sequencer (MGI Tech Co. Ltd, Shenzhen, China). MutMap analysis was performed as previously described (Abe et al. 2012), with *Nipponbare* as the reference genome. Euclidean distance (ED) was calculated as previously described (Hill et al. 2013).

**Total RNA extraction and quantitative real-time PCR**

Total RNA was extracted using the Plant Total RNA Isolation Kit (FOREGENE, Chengdu, China) according to the product manual. Total RNA (500 ng) was used for cDNA synthesis using the RT Easy^TM^ II (with gDNase) (FOREGENE, Chengdu, China). Quantitative real-time PCR (qRT–PCR) was performed on a Bio-Rad CFX96^TM^ real-time system (USA) with ChamQ SYBR qPCR Master Mix (Vazyme, Nanjing, China). *UBQ5* and *FhaB* were used as internal controls (Zhao et al. 2020). Three replicates were performed for each analysis. The primers for qRT–PCR analysis are listed in Additional file 1: Table S2.

**Vector** **construction and plant transformation**

To obtain knockout mutants of *WG3*, the sgRNA target site and primers for construction of the sgRNA expression cassette were designed using the online tools of CRISPR-GE (http://skl.scau.edu.cn/home/) (Xie et al. 2017). The CRISPR/Cas9 vector of *WG3* was generated as previously described (Ma et al. 2015), and was introduced into ZH11 by *Agrobacterium*-mediated transformation. The *WG3*-KO lines were genotyped by PCR amplification and sequencing. Primers used for PCR amplification and vector construction are listed in Additional file 1: Table S3.

**Bimolecular fluorescence complementary (BiFC) assay**

Full-length CDS fragments of *WG3* and *DLT* without stop codons were amplified and cloned into the pSPYNE or pSPYCE vectors (Walter et al. 2004). BiFC assays were performed using the *Nicotiana benthamiana* transient expression system, as previously described (Yuan et al. 2018). The ﬂuorescence was observed and photographed using a confocal microscope (Nikon A1, Tokyo, Japan). The primers for vector construction are listed in Additional file 1: Table S3.

**Lamina inclination assay**

The lamina joint inclination assay was performed as previously described (Yuan et al. 2017). In brief, the lamina joints of the second leaf of R498 and *wg3* mutant plants were treated with 1 µl of 24-epibrassinolide (24-epiBL; 0, 100, 300, and 500 μM). The lamina joint angles were measured using ImageJ software (https://imagej.net/) three days after treatment. Images were taken by a scanner (ScanMaker i800 Plus, Shanghai, China).

**References**

Abe A, Kosugi S, Yoshida K, Natsume S, Takagi H, Kanzaki H, Matsumura H, Yoshida K, Mitsuoka C, Tamiru M, Innan H, Cano L, Kamoun S, Terauchi R (2012) Genome sequencing reveals agronomically important loci in rice using MutMap. Nat Biotechnol 30:174-178

Hill JT, Demarest BL, Bisgrove BW, Gorsi B, Su YC, Yost HJ (2013) MMAPPR: mutation mapping analysis pipeline for pooled RNA-seq. Genome Res 23:687-697

Ma X, Zhang Q, Zhu Q, Liu W, Chen Y, Qiu R, Wang B, Yang Z, Li H, Lin Y, Xie Y, Shen R, Chen S, Wang Z, Chen Y, Guo J, Chen L, Zhao X, Dong Z, Liu Y-G (2015) A Robust CRISPR/Cas9 System for Convenient, High-Efficiency Multiplex Genome Editing in Monocot and Dicot Plants. Mol Plant 8:1274-1284

Tang QY, Zhang CX (2013) Data Processing System (DPS) software with experimental design, statistical analysis and data mining developed for use in entomological research. Insect Sci 20:254-260

Walter M, Chaban C, Schütze K, Batistic O, Weckermann K, Näke C, Blazevic D, Grefen C, Schumacher K, Oecking C, Harter K, Kudla J (2004) Visualization of protein interactions in living plant cells using bimolecular fluorescence complementation. Plant J 40:428-438

Xie X, Ma X, Zhu Q, Zeng D, Li G, Liu YG (2017) CRISPR-GE: A Convenient Software Toolkit for CRISPR-Based Genome Editing. Mol Plant 10:1246-1249

Yuan H, Fan S, Huang J, Zhan S, Wang S, Gao P, Chen W, Tu B, Ma B, Wang Y, Qin P, Li S (2017) 08SG2/OsBAK1 regulates grain size and number, and functions differently in Indica and Japonica backgrounds in rice. Rice (N Y) 10:25

Yuan H, Gao P, Hu X, Yuan M, Xu Z, Jin M, Song W, Zhan S, Zhu X, Tu B, Li T, Wang Y, Ma B, Qin P, Chen W, Li S (2022a) Fine mapping and candidate gene analysis of qGSN5, a novel quantitative trait locus coordinating grain size and grain number in rice. Theor Appl Genet 135:51-64

Yuan H, Xu Z, Chen W, Deng C, Liu Y, Yuan M, Gao P, Shi H, Tu B, Li T, Kang L, Ma B, Wang Y, Wang J, Chen X, Li S, Qin P (2022b) OsBSK2, a putative brassinosteroid-signalling kinase, positively controls grain size in rice. J Exp Bot 73:5529-5542

Yuan M, Xu CY (2018) BiFC Assay for Detecting Protein-protein Interaction in Tobacco Leaves. Bio-protocol:e1010133

Zhao Z, Zhang Z, Ding Z, Meng H, Shen R, Tang H, Liu YG, Chen L (2020) Public-transcriptome-database-assisted selection and validation of reliable reference genes for qRT-PCR in rice. Sci China Life Sci 63:92-101
